# Intrinsic disorder in PRAME and its role in uveal melanoma

**DOI:** 10.1186/s12964-023-01197-y

**Published:** 2023-08-25

**Authors:** Michael Antonietti, David J. Taylor Gonzalez, Mak Djulbegovic, Guy W. Dayhoff, Vladimir N. Uversky, Carol L. Shields, Carol L. Karp

**Affiliations:** 1 Bascom Palmer Eye Institute, University of Miami, 900 NW 17th Street, Miami, FL 33136 USA; 2 Department of Chemistry, College of Art and Sciences, University of South Florida, FL 33612 Tampa, USA; 3 Department of Molecular Medicine and USF Health Byrd Alzheimer’s Research Institute, Morsani College of Medicine, University of South Florida, FL 33612 Tampa, USA; 4 Ocular Oncology Service, Wills Eye Hospital, Thomas Jefferson University, PA Philadelphia, USA

**Keywords:** Uveal melanoma, Intrinsically disordered protein, Disorder-to-order-transition, PRAME, PReferentially expressed Antigen in Melanoma

## Abstract

**Introduction:**

The PReferentially expressed Antigen in MElanoma **(**PRAME) protein has been shown to be an independent biomarker for increased risk of metastasis in Class 1 uveal melanomas (UM). Intrinsically disordered proteins and regions of proteins (IDPs/IDPRs) are proteins that do not have a well-defined three-dimensional structure and have been linked to neoplastic development. Our study aimed to evaluate the presence of intrinsic disorder in PRAME and the role these structureless regions have in PRAME( +) Class 1 UM.

**Methods:**

A bioinformatics study to characterize PRAME’s propensity for the intrinsic disorder. We first used the AlphaFold tool to qualitatively assess the protein structure of PRAME. Then we used the Compositional Profiler and a set of per-residue intrinsic disorder predictors to quantify the intrinsic disorder. The Database of Disordered Protein Prediction (D^2^P^2^) platform, IUPred, FuzDrop, fIDPnn, AUCpred, SPOT-Disorder2, and metapredict V2 allowed us to evaluate the potential functional disorder of PRAME. Additionally, we used the Search Tool for the Retrieval of Interacting Genes (STRING) to analyze PRAME's potential interactions with other proteins.

**Results:**

Our structural analysis showed that PRAME contains intrinsically disordered protein regions (IDPRs), which are structureless and flexible. We found that PRAME is significantly enriched with serine (*p*-value < 0.05), a disorder-promoting amino acid. PRAME was found to have an average disorder score of 16.49% (i.e., moderately disordered) across six per-residue intrinsic disorder predictors. Our IUPred analysis revealed the presence of disorder-to-order transition (DOT) regions in PRAME near the C-terminus of the protein (residues 475–509). The D^2^P^2^ platform predicted a region from approximately 140 and 175 to be highly concentrated with post-translational modifications (PTMs). FuzDrop predicted the PTM hot spot of PRAME to be a droplet-promoting region and an aggregation hotspot. Finally, our analysis using the STRING tool revealed that PRAME has significantly more interactions with other proteins than expected for randomly selected proteins of the same size, with the ability to interact with 84 different partners (STRING analysis result: *p*-value < 1.0 × 10^–16^; model confidence: 0.400).

**Conclusion:**

Our study revealed that PRAME has IDPRs that are possibly linked to its functionality in the context of Class 1 UM. The regions of functionality (i.e., DOT regions, PTM sites, droplet-promoting regions, and aggregation hotspots) are localized to regions of high levels of disorder. PRAME has a complex protein–protein interaction (PPI) network that may be secondary to the structureless features of the polypeptide. Our findings contribute to our understanding of UM and suggest that IDPRs and DOT regions in PRAME may be targeted in developing new therapies for this aggressive cancer.

Video Abstract

**Supplementary Information:**

The online version contains supplementary material available at 10.1186/s12964-023-01197-y.

## Introduction

Uveal melanoma (UM) is the most common primary intraocular tumor [1]. The neoplasm most commonly arises from the choroid but also develops in the ciliary body (6%) and iris (4%) [1]. UM affects both men and women equally, and it most commonly affects middle-aged White individuals with a median age of 58 years [2]. Though there have been advances in the detection, prognostication, and treatment of UM, the risk for metastasis is high (50%) and is often associated with poor outcomes [1, 3].

Gene expression profiling (GEP) has been developed to assess the risk of metastasis for UM. GEP allows clinicians to group UM into either Class 1 (low-grade metastatic risk) or Class 2 (high-grade metastatic risk) based on their molecular profiles [4]. Class 1 UM is associated with upregulation of PReferentially expressed Antigen in MElanoma (PRAME), and class 2 UM mutations are associated with mutations or biallelic inactivation of BRCA1 (breast cancer type 1 susceptibility protein)-associated protein 1 (BAP1) [5–8]. The 5-year actuarial rate for metastasis of class 1 PRAME(-), class 1 PRAME( +), class 2 tumors is 0%, 38%, and 71% respectively [9]. These numbers suggest that PRAME may serve as an independent biomarker for the risk of metastasis in Class 1 UM.

Our group has studied ocular malignancies and characterized proteins involved in ocular surface squamous neoplasia, conjunctival melanoma, and uveal melanoma [8]. We are interested in the intrinsic disorder phenomenon (i.e., proteins or regions of proteins that have no definitive 3D structure) and how it may play a role in the pathogenesis of ocular neoplasms [8, 10]. We previously demonstrated that BAP1 has elements of intrinsic disorder [8]. Dysregulation of these structureless regions secondary to BAP1 mutations may promote metastatic behavior in Class 2 UM. This study recapitulates our previous analysis and applies it to Class 1 UM and PRAME. While there are many interactions to consider when looking at the role of PRAME in the tumor pathogenesis of UM, our group wanted to understand PRAME’s intrinsic disorder propensity and its potential to provide a framework for developing biologics or immunotherapies to target PRAME.

Intrinsically disordered proteins (IDPs) and intrinsically disordered protein regions (IDPRs) are proteins and the regions of proteins that do not adopt a well-defined three-dimensional structure and instead are disordered but maintain function [11]. These functional proteins or protein regions are found in all known proteomes and do not adopt a tertiary structure [12]. IDPs and IDPRs have been linked extensively to the pathogenesis of disease and malignancies [13–17]. In this study, we aim to evaluate the intrinsic disorder of PRAME and its role in pathogenesis and driving neoplastic development in Class 1 UM. Furthermore, the IDPRs of PRAME may serve as targets for treating PRAME( +) UM, thereby decreasing the risk of malignancy of the primary tumor in those most vulnerable.

## Methods

Our study focused on the intrinsic disorder phenomenon and its association with PRAME expression and its association with uveal melanoma. To accomplish this, we conducted a disorder-based bioinformatics analysis that utilized publicly available databases and computational tools to probe PRAME for intrinsically disordered protein regions.

### Protein sequences

The Universal Protein Resource (UniProt) is a protein database with information related to function, taxonomy, subcellular location, disease associations, post-translation modifications, expression profiles, interaction networks, structure, protein families, and sequences (available at: https://uniprot.org; date last accessed: Dec 26, 2022) [18]. The search term used was “protein: PRAME.” The human variant was selected for PRAME (UniProtID: P78395). The default (canonical) amino acid sequence was selected for further analysis.

### Structural assessment

The UniProt entries for PRAME also contain structural information. There are computational and experimental-based structures (i.e., nuclear magnetic resonance, cryo-electron microscopy, and x-ray crystallography) housed on the UniProt server. We selected the “AlphaFoldDB” entry. AlphaFold is the most up-to-date and accurate computational method to predict protein structure [19–21].

### Quantification of intrinsic disorder

#### Compositional profiler

We sought to quantify the elements of intrinsic disorder in PRAME. We utilized the Compositional Profiler to measure the relative proportion of specific amino acids in each protein (available at: http://www.cprofiler.org/; accessed on December 26, 2022) [22]. Each amino acid was compared to a background set of proteins (i.e., Protein Data Bank Select (PBD) 25 [22]) that are a standard set of highly ordered proteins (i.e., proteins that contain many elements of structure in the form of alpha helices or beta-pleated sheets) [22]. We evaluated the normalized enrichment or depletion of each amino acid as $\frac{{C}_{x- }{C}_{order}}{{C}_{order}}$, where *C*_*x*_ is the content of a given residue in a query protein (i.e., PRAME) and *C*_*order*_ is the content of the same residue in the PDB Select 25.

#### Per-residue disorder prediction

The intrinsic disorder of PRAME was quantified on a per-residue basis using a set of ten well-established disorder predictors, namely: PONDR® VLS2 [23, 24], PONDR® VL3 [25], PONDR® VLXT [26], PONDR® FIT [27], IUPred-Long, IUPred-Short [28], flDPnn [29], AUCpreD [30], SPOT-Disorder2 [31] and metapredict V2 [32]. The Rapid Intrinsic Disorder Analysis Online (RIDAO) platform (https://ridao.app) was used to yield bulk predictions for the PONDR family of predictors (i.e. VLS2, VL3, VLXT, and FIT) as well as IUPred-Long and IUPred-Short. The remaining predictions were obtained using local installations of the respective predictors—flDPnn, AUCpreD, SPOT-Disorder2, and metapredict V2. Each predictor used in this work takes a primary sequence as input and yields a measure of intrinsic disorder propensity, referred to as the disorder score, for each amino acid contained therein. Disorder scores range from 0 to 1, where 0 is the most ordered measurement and 1 is the most disordered. Taken as a whole, the complete set of disorder scores for a given sequence constitute a disorder profile.

Using the disorder profile of PRAME we computed the percent of predicted disordered residues (PPDR) for a given predictor, $p$, as,

1 $${PPDR}_{p}= 100*\frac{{N}_{disordered}}{{N}_{total}}$$

where ${N}_{disordered}$ is the number of residues with a disorder score greater than or equal to 0.5 (or 0.3 in the case of flDPnn) and ${N}_{total}$is the total number of residues in the sequence. A protein is classified as highly ordered when the PPDR is less than 10%, highly disordered when the PPDR is greater than 30%, and moderately disordered in all other cases [33]. Furthermore, we computed the average disorder score (ADS) over a range of residues for a given predictor, $p$, as,

2 $${ADS}_{p}=\frac{\sum_{r=a}^{b}{x}_{r}}{b-a}$$

where $a$ is the first residue to consider, $b$ is the last residue to consider, and ${x}_{r}$ is the disorder score of residue $r$, given by predictor $p$. A protein or protein region is characterized as ordered when the ADS is less than 0.15, disordered when the ADS is equal to or greater than 0.5, and moderately disordered, or flexible, in all other cases. Importantly, we do not compute the ADS for the flDPnn predictor for which a value greater than or equal to 0.3 is used to identify disordered residues.

### Assessment of disorder-related functionality

#### Analyzing functionality using D^2^P^2^ platform

Disorder-related functionality of PRAME was evaluated with the Database of Disordered Protein Prediction (D^2^P^2^) platform (http://d2p2.pro/; accessed on November 17, 2022) [34]. The D^2^P^2^ platform integrates IUPred [35], PONDR® VLXT [26], PONDR® VSL2B [23, 24], PrDOS [36], PV2 [34], and ESpritz [37], allowing for a consolidated visualization of disorder prediction tools. The D^2^P^2^ server provides the Structural Classification of Proteins (SCOP) [38, 39], SUPERFAMILY predictor [40], and InterPro prediction for the sequences of interest (https://www.ebi.ac.uk/interpro/search/sequence/, accessed on December 12, 2022) [41]. D^2^P^2^also predicts disorder-based binding sites with the ANCHOR algorithm [42]. ANCHOR-identified disordered protein-binding regions (DPBRs) that include short binding regions, which are disordered in the unbound form and expected to fold into ordered structures when interacting with specific partners (i.e., molecular recognition features (MoRFs) and other disordered protein binding domains that are longer than 25 residues [43]. Finally, the D^2^P^2^platform also predicts possible posttranslational modifications (PTMs) using the outputs of the PhosphoSitePlus [44].

#### Identification of redox-sensitive disorder-to-order transition (DOT) regions

The IUPred3 platform was the final step in quantifying intrinsic disorder within PRAME (available at: https://iupred3.elte.hu/; accessed on November 17, 2022) [28, 45, 46]. This tool can assess the location of disordered redox-sensitive protein regions in PRAME by determining the disorder score in both their disordered and ordered state. If the values vary significantly between the redox states, we can define a particular region as a redox-sensitive region (i.e., a disorder-to-order transition (DOT) region).

#### Identification of droplet-promoting regions (DPRs), aggregation hot spots, and regions with cellular context-dependent interactions

Additional functional information on human PRAME was retrieved using the FuzDrop platform (https://fuzdrop.bio.unipd.it/predictor; accessed on December 27, 2022) [47]. This tool provides information on the capability of a protein to undergo liquid–liquid phase separation, identifies the presence and localization of droplet-promoting regions (DPRs), shows the location of aggregation hot-spots (i.e., residues/regions that may promote the conversion of a protein to a liquid-like condensed state into a solid-like amyloid state) [48, 49]. FuzDrop also provides the probability of a protein having residues/regions with cellular context dependence (i.e., those characterized by the ability of residues to switch between different binding modes) [48].

### Protein–protein interaction network

To further understand the protein–protein interactions of PRAME, the amino acid sequences were used as inputs for The Search Tool for the Retrieval of Interacting Genes (STRING) (URL: https://string-db.org/; accessed on November 17, 2022). STRING is a quality-controlled database that utilizes experimentally and computationally derived data to detail functional interactions between proteins [50]. The minimum required interaction score settings were set to the highest confidence (0.900), and the maximum number of interactions was assigned the highest possible value of 500.

## Results

### Protein sequence

Our disorder-based bioinformatics protein analysis used the canonical amino acid sequence of PRAME in FASTA format. PRAME contains 509 amino acid residues and includes nine leucine-rich repeats (LLR) at residues 116–145, 207–231, 232–258, 259–294, 295–320, 321–352, 353–371, 377–404, and 405–429. There is also a region (residues 416–509) that mediates the interaction with a retinoic acid receptor alpha (RARA).

### Structural assessment

**Fig. 1:**
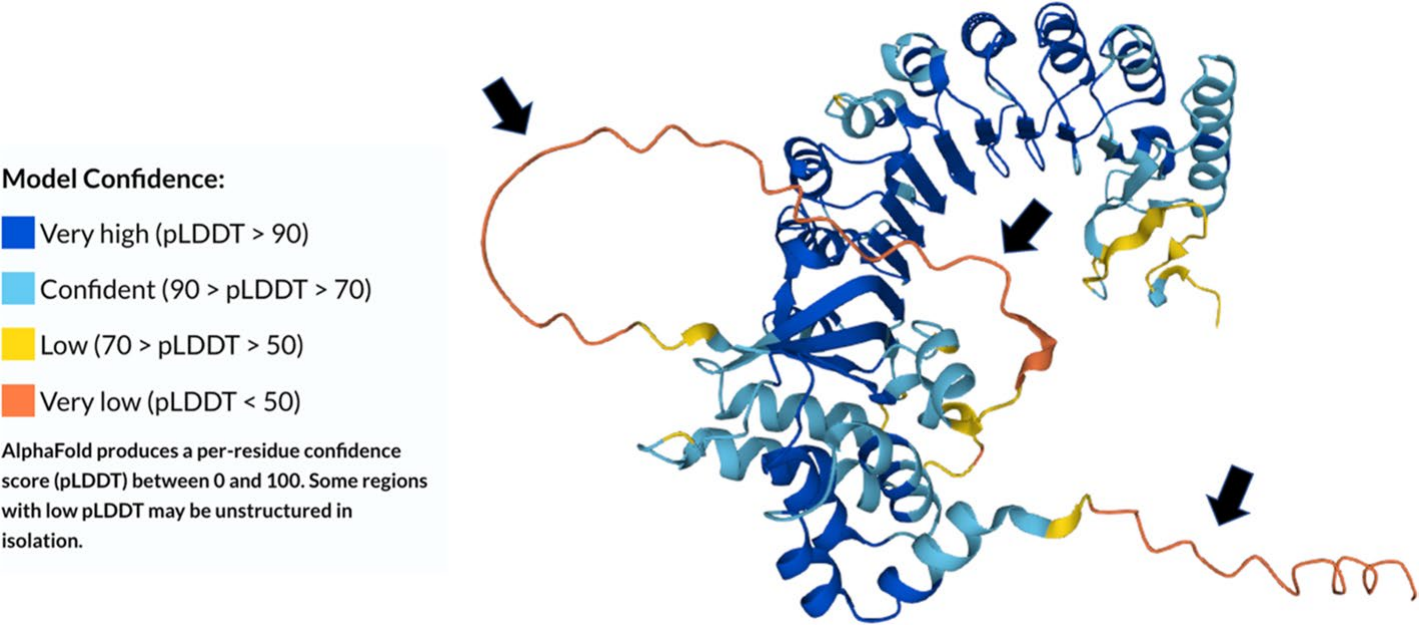
Structures generated for PRAME by AlphaFold2. As measured by the predicted local distance difference test (pLDDT), regions with higher model confidence typically represent alpha-helices and beta-pleated sheets. Regions with lower levels of model confidence might represent intrinsically disordered protein regions. The black arrows indicate areas of potentially high levels of disorder that lack definitive structure

The 3D structure of PRAME modeled by AlphaFold (Fig. 1) demonstrates that PRAME has both elements of order (structure; α-helices and β-pleated sheets) and disorder (structureless, spaghetti-like regions) (Fig. 1). Structural portions of the protein are flanked by regions that lack definitive structure (residues 1–23 and 138–172). The C-terminal region of PRAME (residues 487–509) is also predicted to have low model confidence (i.e., high intrinsic disorder). Figure 1 shows that the AlphaFold model confidence is high for a large proportion of PRAME’s structure, as indicated by the extensive blue color throughout the model. The terminals and disorder regions of PRAME appear to be less ordered, as indicated by low model confidence and yellow/orange coloring. This reflects the regions of PRAME with a less established structure. These regions of PRAME with less model confidence are the intrinsically disordered protein regions (IDPRs) of interest. From a qualitative point of view, it is evident that there are segments in PRAME that contain little to no structure yet likely retain biological functions as expected for most IDPRs.

### Quantification of intrinsic disorder

#### Compositional profiler

**Fig. 2:**
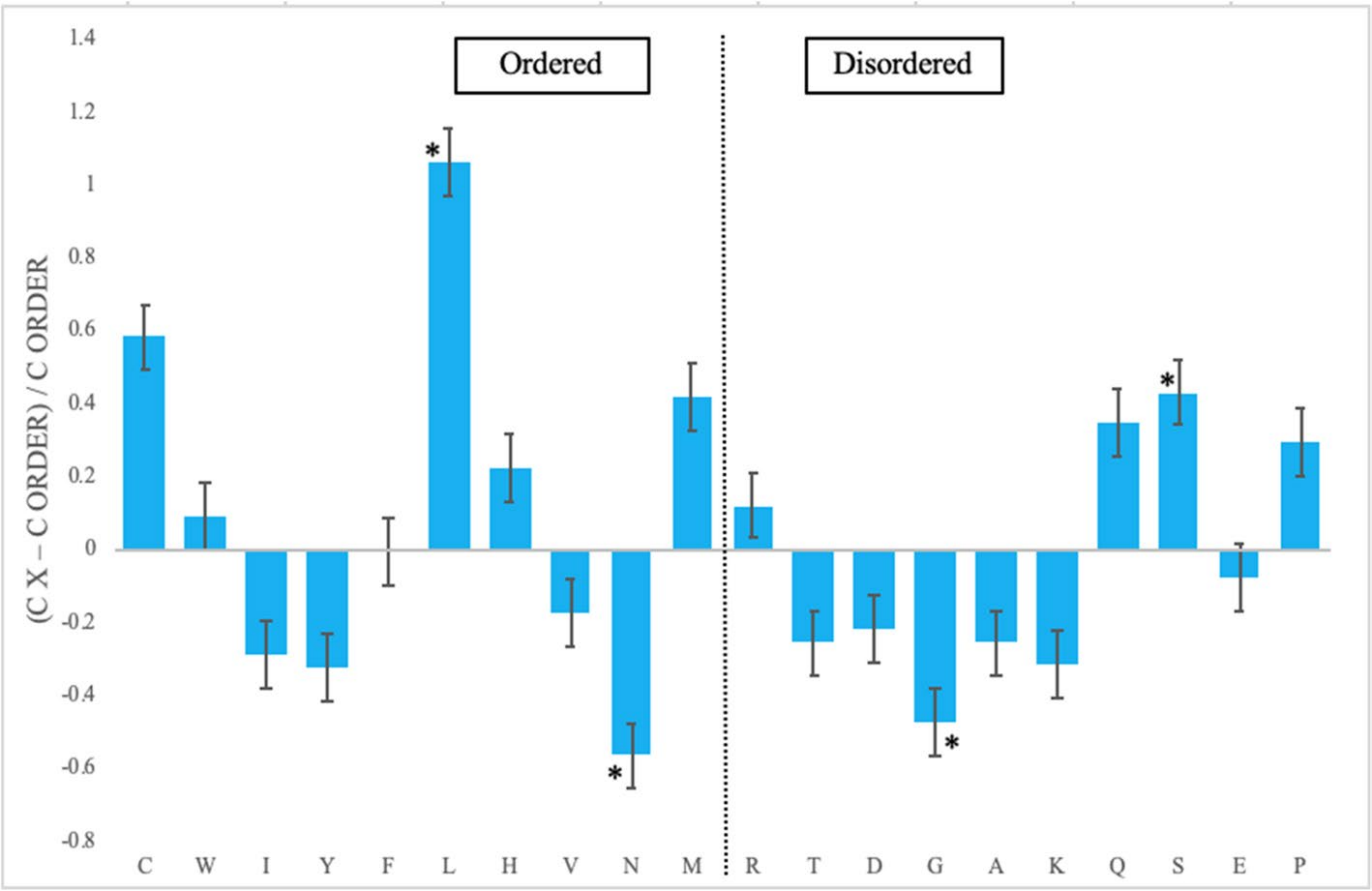
Amino acid composition profile of PRAME. The fractional difference is calculated as $\frac{{C}_{x- }{C}_{order}}{{C}_{order}}$, where *C*_*x*_ is the content of a given amino acid in the query set (PRAME), and *C*_*order*_ is the content of a given amino acid in the background set (Protein Databank Select 25). Amino acids marked with (*) are statistically significant for enrichment (L and S) or depletion (G and N) (*p*-value < 0.05). The amino acid residues are ranked from most order promoting residues ((i.e., cysteine (C), tryptophan (W), isoleucine (I), tyrosine (Y), phenylalanine (F), leucine (L), histidine (H), valine (V), asparagine (N), and methionine (M)) to most disorder promoting residues ((i.e., arginine (R), threonine (T), aspartate (D), glycine (G), alanine (A), lysine (K), glutamine (Q), serine (S), glutamate (E), and proline (P)). Positive values indicate enrichment, and negative values indicate depletion of amino acids

After visualization of the IDPRs in PRAME within the AlphaFold-modeled structure, we aimed to quantify these regions. We first constructed a normalized amino acid composition profile for PRAME (Fig. 2). In the composition profile, each amino acid was arranged from the most order-promoting (i.e., cysteine (C), tryptophan (W), isoleucine (I), tyrosine (Y), phenylalanine (F), leucine (L), histidine (H), valine (V), asparagine (N), and methionine (M)) to the most disorder-promoting residues (i.e., arginine (R), threonine (T), aspartate (D), glycine (G), alanine (A), lysine (K), glutamine (Q), serine (S), glutamate (E), and proline (P)). If the amino acid is enriched in the protein, then it receives a positive value. If the amino acid is depleted in the protein, then it receives a negative value. PRAME is significantly enriched in one order-promoting amino acid (L) and significantly depleted in one disorder-promoting amino acid (N). PRAME is significantly enriched in one disorder-promoting residue (S) and significantly depleted in (G).

#### Per-residue disorder prediction

**Fig. 3:**
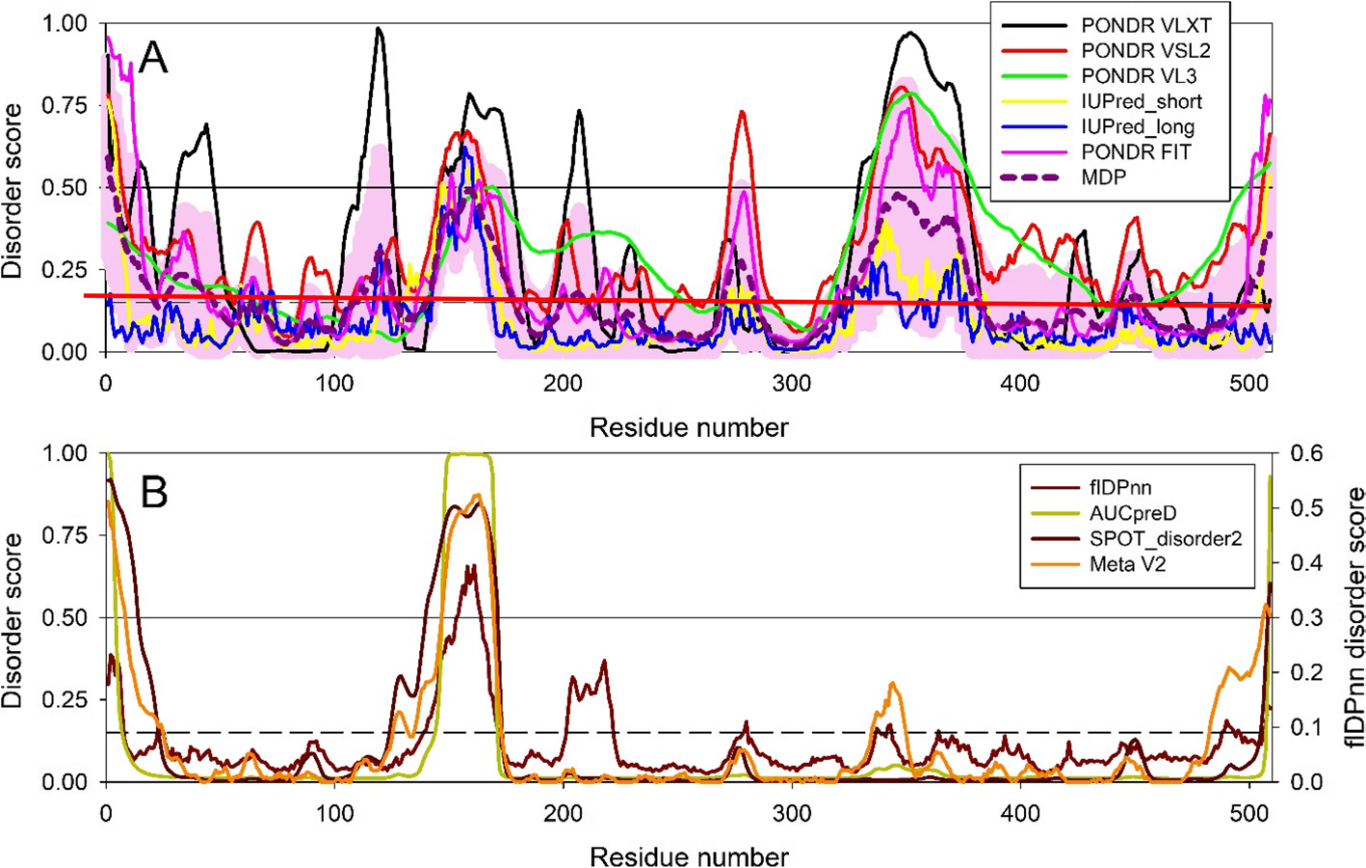
Evaluation of the intrinsic disorder predisposition of PRAME. A. Per-residue intrinsic disorder profiles generated by RIDAO that assembles the outputs of PONDR® VLXT, PONDR® VL3, PONDR® VSL2, PONDR® FIT, IUPred_long and IUPred_short. The mean disorder profile (MDP) of the protein is calculated by averaging the disorder profiles of individual predictors. The light pink shade represents the MDP error distribution. The thin black line at the disorder score of 0.5 is the threshold between order and disorder, where residues/regions above 0.5 are disordered, and residues/regions below 0.5 are ordered. The solid red line at the disorder score of 0.15 is the threshold between order and flexibility, where residues/regions above 0.15 are flexible, and residues/regions below 0.15 are highly ordered. B. Per-residue intrinsic disorder profiles generated by predictors that performed better at a biannual blind test, Critical Assessment of protein Intrinsic Disorder prediction (CAID) [52]. These predictors are fIDPnn [53], AUCpreD [54], and SPOT-Disorder2 [31]. We also included the outputs of the Metapredict V2, which is a deep-learning-based predictor of consensus disorder scores [32]. Note that most predictors used in this study utilize the threshold of 0.5 to discriminate between ordered and disordered residues. The only exception is given by fIDPnn that utilizes the threshold of 0.3 for the same purpose (see right-hand Y axis in the plot B). Figure shows that PRAME is largely ordered protein but possesses elements of disorder throughout (i.e., regions comprising residues 1–15, 136–177, 330–376, and 510–509 are predicted as disordered by several predictors)

After determining the amino acid composition of PRAME, we aimed to quantify the intrinsic disorder on a per-residue basis. We used PONDR® VLXT [26], PONDR® VL3, PONDR® VSL2, and PONDR® FIT, as well as IUPred-Long and IUPred-Short to predict the disorder score of each amino acid in PRAME (Fig. 3A). The six disorder predictors that make up the RIDAO suite [51] allow for the classification of proteins and protein regions using the established criteria for the classification of proteins based on their percent of the predicted disordered residues (PPDR scores), where proteins are considered highly ordered (PPDR < 10%), moderately disordered (10% ≤ PPDR < 30%), and highly disordered (PPDR ≥ 30%) [22]. PRAME was predicted to be 28.36% disordered by PONDR® VLXT (i.e., moderately disordered), 28.02% disordered by PONDR® VL3 (i.e., moderately disordered), 30.89% disordered by PONDR® VSL2 (i.e., highly disordered), 20.61 disordered by PONDR-FIT (i.e., moderately disordered), 10.31% disordered by IUPred-Long (i.e., moderately disordered), and 9.39% disordered by IUPred-Short (i.e., highly ordered). To provide further validation of the presence of intrinsic disorder in the PRAME protein, we used a set of predictors that showed good performance at a biannual blind test [52]. These predictors are fIDPnn [53], AUCpreD [54], and SPOT-Disorder2 [31]. We also utilized a deep-learning-based predictor of consensus disorder scores, metapredict V2 [32]. The corresponding data are shown in Fig. 3B illustrating that at least three regions of PRAME (residues 1–15, 140–172, and 505–509) are predicted as disordered by some of these tools as well. Notably, PONDR® VSL2, IUPred-Long and IUPred-Short also participated in the CAID competition, where they performed reasonably well [52]. Furthermore, we utiliized flDPnn to predict both the intrinsic disorder status of PRAME and also predicts its disorder-based functions [53]. We utilized this tool to look for the presence of disorder-based protein-, DNA- and RNA-biding regions. This analysis revealed that the intrinsically disordered region comprising residues 154–164 can be engaged in interactions with proteins, DNA and RNA.

### Assessment of disorder-related functionality

#### Analyzing functionality using D^2^P^2^ platform

**Fig. 4:**
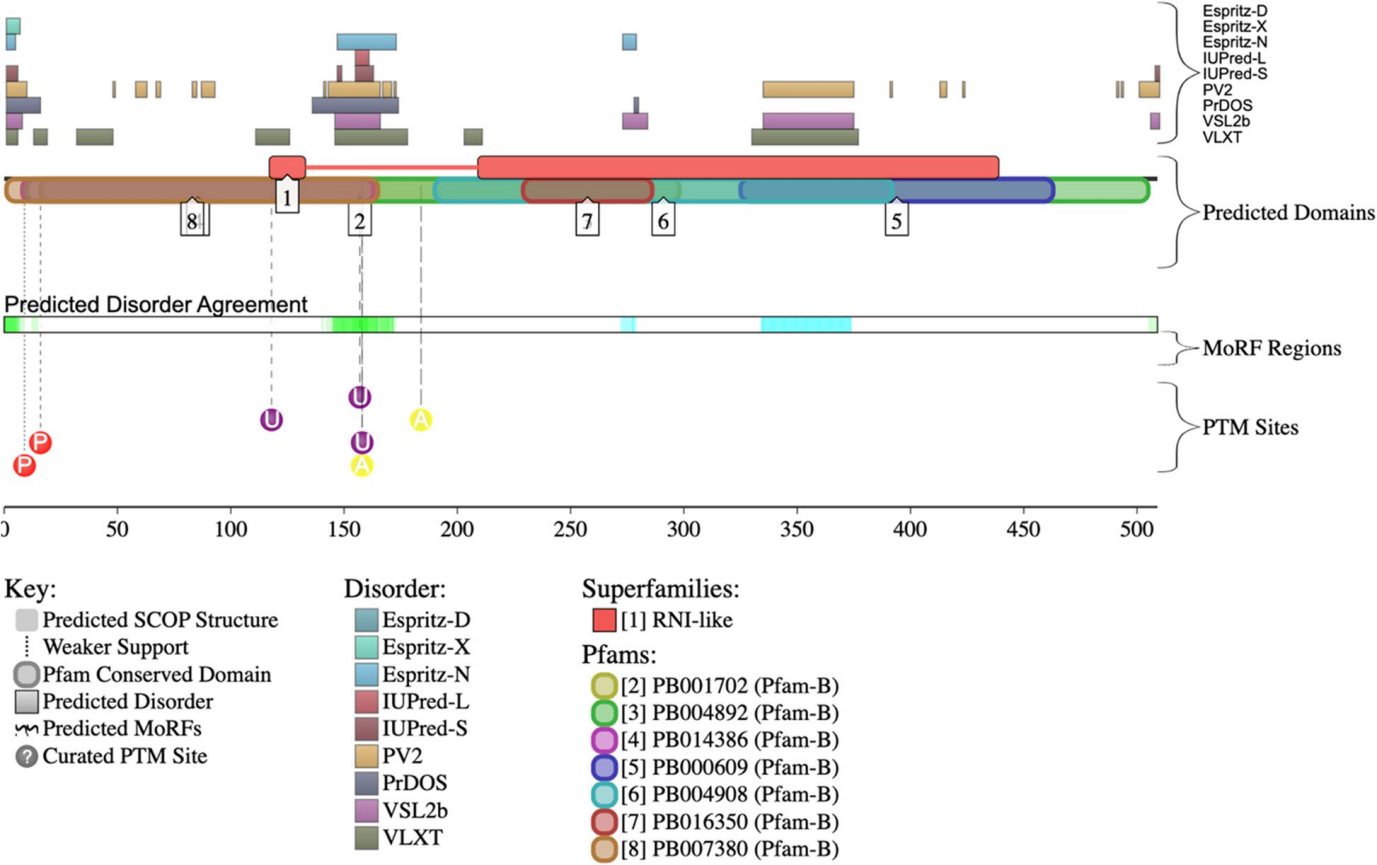
The D^2^P^2^ platform output assesses the functional disorder profile for PRAME. On the figure's left are four identifiers for predictors used on the D^2^P^2^ platform. The top right of the figure shows outputs from various per-residue disorder predictors (i.e., Espritz-D, Espritz-X, Espritz-N, IUPred-L, IUPred-S, PV2, PrDOS, VSL2b, VLXT); the second bracket shows predicted protein domains, the third bracket shows where molecular recognition features are located (MoRFs; i.e., disorder regions that become ordered when binding), and the fourth bracket shows posttranslational modifications (PTM) sites. The protein domains fall within regions predicted to be ordered or disordered. The MoRF regions are localized to regions that demonstrate extensive disorder. The PTMs are also predominantly localized to areas of each protein that contain intrinsic disorder

To assess the functionality of the intrinsic disorder in PRAME, we used the D^2^P^2^ platform (Fig. 4). The D^2^P^2^ platform allows quantification of the functional intrinsic disorder of each protein and assess its relationship with post-translational modifications (PTMs) and disorder-based binding potential. PRAME’s D^2^P^2^ output (Fig. 4) demonstrates relative agreement between the nine disorder-based predictors, where scattered regions of disorder predicted throughout the protein are present. The region of PRAME that demonstrates the most agreement among the nine per-residue disorder predictors is approximately from amino acid region 130 to 160. PRAME contains several conserved functional domains. There were two RNI like domains (residue 118–133, 210–439) identified in the SUPERFAMILY database, and the InterPro server identified domains; PB007380 (residues 2–165), PB001702 (residues 120–248), PB004892 (residues 11–505), PB014386 (residues 1–165), PB000609 (residues 326–463), PB004908 (residues 191–392), and PB016350 (residues 230–286). The InterPro server places the human PRAME into the PRAME family (InterPro ID: IPR026271). LLR-containing domain (residues 188–469) of this protein is a member of the leucine-rich repeat domain superfamily (InterPro ID: IPR032675). InterPro also identifies this region as a ribonuclease inhibitor by CATH-Gene3D, where part of this region (residues 188–443) is included in Functional Families (InterPro ID: FF000354). Finally, according to the InterPro annotations, human PRAME is expected to be involved in the negative regulation of the apoptotic process (GO:0043066), positive regulation of cell population proliferation (GO:0008284), negative regulation of DNA-templated transcription (GO:0045892), and negative regulation of cell differentiation (GO:0045596). There are no molecular recognition features (MoRFs) in PRAME as predicated by the D^2^P^2^ platform. PRAME has 7 predicted post-translational modifications (PTMs), 3 ubiquitination sites, 2 acetylation sites, and 2 phosphorylation sites.

#### Identification of redox-sensitive disorder-to-order transition (DOT) regions

**Fig. 5:**
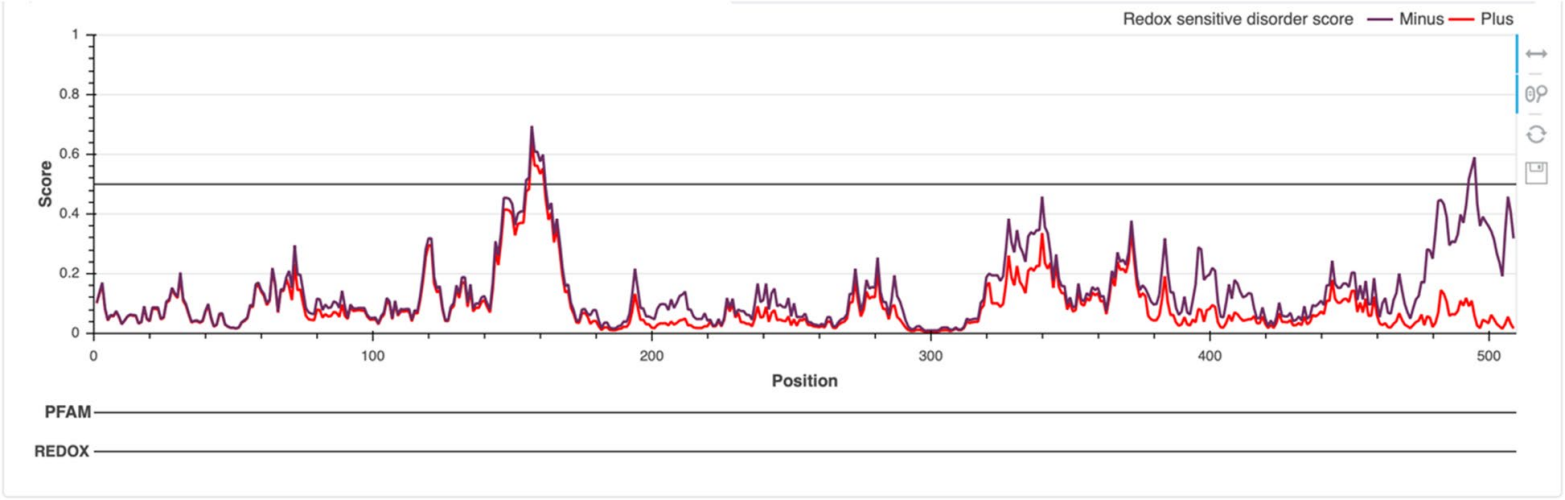
Evaluation of redox-sensitive disorder predispositions of PRAME by IUPred2A_redox. The corresponding plot shows the disorder profile for the reduced (redox-minus) and oxidized (redox-plus) forms of a protein by violet and red lines, respectively. Shaded areas (not seen in PRAME) represent significant redox-sensitive regions. The C-terminus of PRAME (region is from amino acids 475 to 509) is a non-significant redox-sensitive, conditionally disordered/flexible region (i.e., a region that undergoes disorder-to-order transition (DOT) in a redox-sensitive manner). In its reduced state (redox-minus), the C-terminus is not highly disordered but flexible, characterized by an IUPred average disorder score (ADS^IUPred^_redox_) of 0.35 ± 0.11. In its reduced state (redox-plus), the C-terminus region is highly ordered (ADS.^IUPred^_redox_ = 0.059 ± 0.035)

We next sought to understand further PRAME’s ability to under disorder-to-order transition (DOT). To assess whether this protein can undergo conformational reorganization following changes in the redox state of its environment (i.e., regions potentially capable of interaction with binding partners in a redox-sensitive manner), the IUPred’s redox tool was utilized. We identified one segment of PRAME that is predicted to be moderately disordered in the reduced state and undergoes a transition to a highly ordered state in the oxidized form (i.e., DOT). This redox-related DOT of PRAME is near the C-terminus of the protein (i.e., residues 475–509) (Fig. 5). This region was marked by high levels of disorder, as evidenced by our per-residue disorder analysis (see Fig. 3).

#### Identification of droplet-promoting regions (DPRs), aggregation hot-spots, and regions with cellular context-dependent interactions

**Fig. 6:**
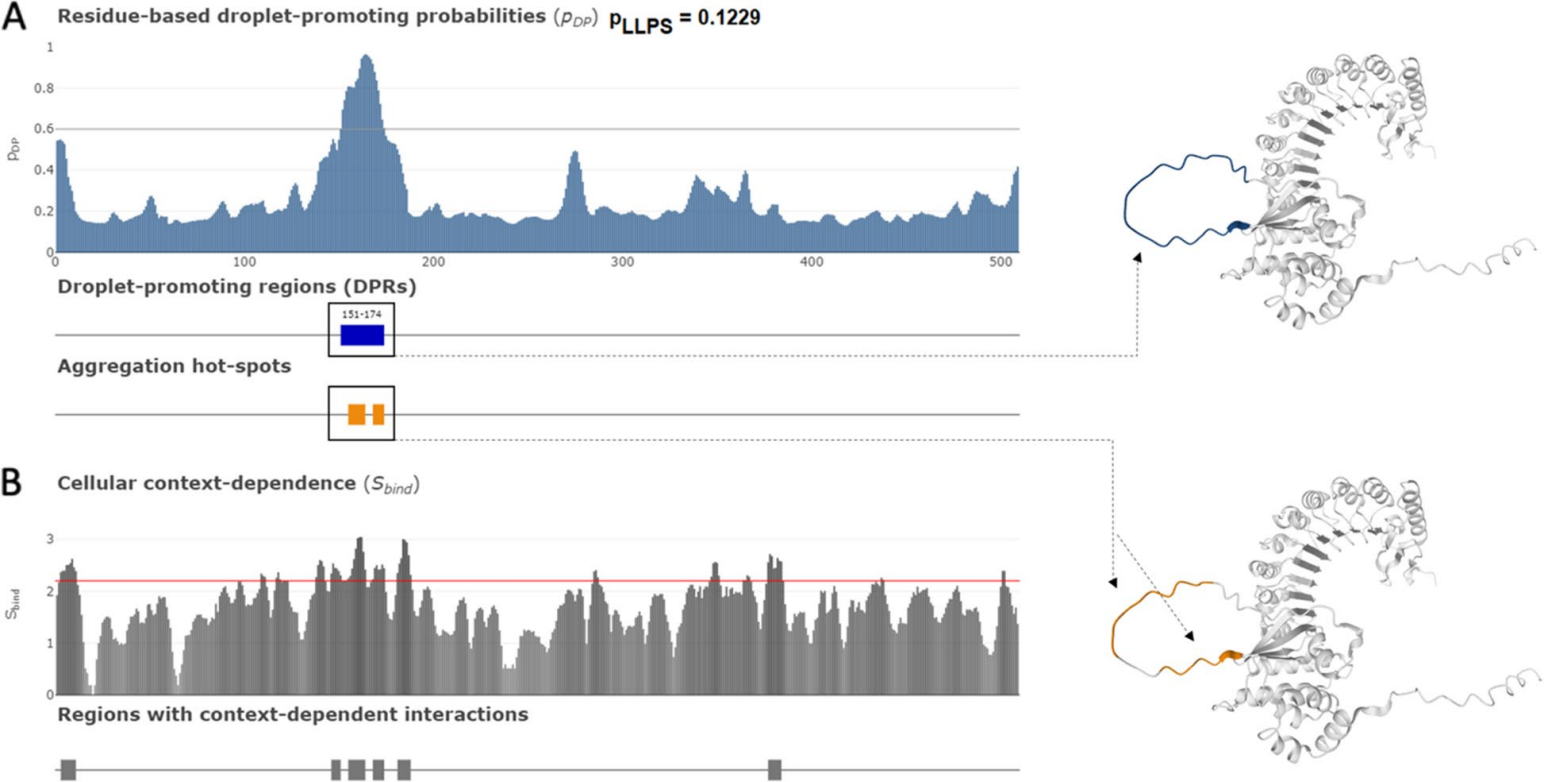
Evaluation of liquid–liquid phase separation propensity of human PRAME by FuzDrop. **A** PRAME has an overall p_LLPS_ of 0.1229, indicating a low probability of promoting droplet formation or liquid–liquid phase separation. However, the protein does contain one region that promotes droplet formation, as depicted in the top AlphaFold structure, as well as two hotspots that promote aggregation, as shown in the bottom AlphaFold structure. **B** Furthermore, the behavior of PRAME is influenced by its cellular context, as indicated by the S_BIND_ value, which describe the ability of specific residues to switch between different binding modes. Regions that exhibit context-dependent interactions can alter the protein's binding behavior and binding modes in various cellular conditions. Residues or regions with S_BIND_ values of 2.25 or greater are capable of transitioning between disordered and ordered states

The propensity of a given protein to undergo liquid–liquid phase separation (LLPS) can be evaluated computationally with FuzDrop [47]. One of the advantages of this tool is its capability to group proteins based on their LLPS propensity (p_LLPS_), where proteins capable of spontaneous LLPS are classified as droplet-driving proteins, whereas proteins that need some additional interactions to form droplets are classified as clients. As per FuzDrop, LLPS drivers are proteins with the overall p_LLPS_ ≥ 0.60, whereas proteins with lower overall p_LLPS_ but containing droplet-promoting regions (DPRs) defined as consecutive residues with p_LLPS_≥ 0.60 will likely serve as droplet-clients [47]. Fig. 6 shows that although PRAME is characterized by a low LLPS propensity of 0.1229, it contains one DPR (residues 151–174). Therefore, although PRAME cannot phase separate by itself, it can serve as a droplet client. Importantly, the DPR of PRAME represents a part of its long internal IDPR (residues 146–177).

Another important advantage of FuzDrop is its capability to find aggregation hot-spots, defined as residues/regions that may promote the conversion of the liquid-like condensed state into a solid-like amyloid state, capable of undergoing both ordered and disordered binding modes that can drive this process [48, 49]. Fig. 6 shows that human PRAME contains two of these hot-spots (residues 155–164 and 168–174).

Finally, FuzDrop provides an outlook of the probability of a protein having residues/regions with cellular context-dependence (i.e., regions containing residues characterized by the ability to switch between disorder and order-based binding) [48]. Fig. 6 demonstrates that PRAME contains six regions with context-dependent interactions (residues 3–11, 146–151, 155–164, 168–174, 181–188, and 377–384). Importantly, these regions with context-dependent interactions overlap with, are included in, or are located in close proximity to the IDPRs found in PRAME (e.g., residues 1–23, 146–176, and 330–376). Furthermore, the most N-terminal disordered region with the context-dependent interactions also includes two phosphorylation sites (Ser^9^ and Ser^16^), suggesting that the functionality of this region can be regulated by posttranslational modifications.

### Protein–protein interaction network

**Fig. 7:**
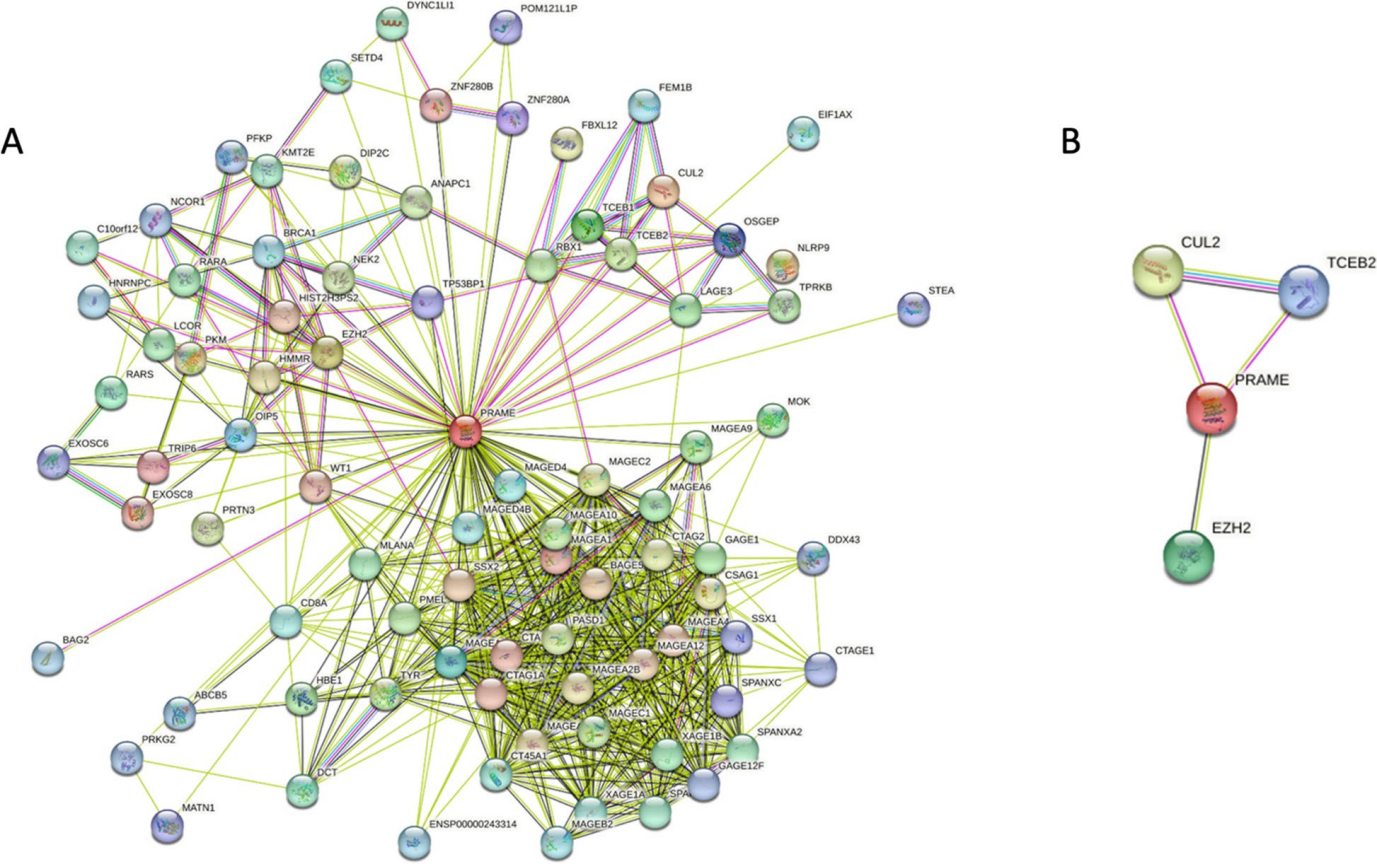
First shell interaction network of PRAME as determined by The Search Tool for the Retrieval of Interacting Genes (STRING). **A** The STRING model confidence was set to medium (0.400). There are 85 nodes in the network with 577 edges (i.e., interactions). The number of interactions was significantly more than the expected 126 interactions in a randomly selected set of proteins of the same size (*p*-value < 1.0 × 10^–16^). **B** The STRING model confidence was set to high (0.900). There were 4 nodes in the network with 4 edges (interactions). The number of interactions was approximately the same as what was expected for a randomly selected set of proteins of the same size (*p*-value < 0.337)

Our analysis has elucidated that intrinsic disorder is embedded into the structure of PRAME. The last step in our disorder-based bioinformatics analysis was focused on understanding how the intrinsic disorder may apply to the binding capabilities of PRAME. Our STRING analysis demonstrated that PRAME has multiple binding partners (Fig. 7). Figure 7A demonstrates PRAME’s STRING network with medium model confidence (0.400), which shows 85 nodes in the network with 577 edges (interactions) and has significantly more than the 126 expected interactions in a randomly selected set of proteins the same size (*p*-value < 1.0 × 10^–16^). At a STRING model confidence of high (0.900, Fig. 7B), there are 4 predicted nodes in the 1^st^ shell interaction network. There are 4 predicted edges (i.e., interactions) in the network, and the number of interactions was approximately the same as what was expected for a randomly selected set of proteins of the same size (*p*-value < 0.337).

## Discussion

To the best of our knowledge, this work is the first to elucidate the presence of potentially functional intrinsically disordered protein regions (IDPRs) in PRAME. Our findings reveal the intrinsically disordered protein segments and regions where disorder is present within PRAME. This highlights the potential role these regions have in PRAME’s function that could be a possible focus for the development of immunotherapies. We show here that PRAME has several IDPs, some of which are relatively long. Furthermore, we demonstrate that these structureless regions are associated with dynamic portions of the protein and that PRAME has a complex PPI network. Our findings must be considered when evaluating the underlying molecular processes that drive the biological behavior of PRAME( +) Class 1 uveal melanoma.

The AlphaFold model of PRAME qualitatively demonstrated the presence of several IDPRs (i.e., spaghetti-like regions). Our quantitative analysis indicated that PRAME was most enriched with leucine (*p*-value < 0.05), which is partly due to the presence of nine leucine-rich repeats embedded in its sequence. Of the disorder-promoting residues, PRAME was significantly enriched with serine (*p*-value < 0.05). Note that at least two of those (Ser^9^ and Ser^16^) were also identified as phosphorylation sites, indicating that they are likely functional hotspots for the protein. PRAME’s average disordered score was 16.49%, classifying it as a moderately disordered and flexible protein.

Additionally, there are seven predicted post-translational modifications (PTMs) in PRAME. These amino acid sites are vital to the protein’s function and are concentrated in areas of high levels of disorder (i.e., the N-terminus and amino acid region 140 to 160). The concentration of PTMs in intrinsically disordered regions are known to produce a range of regional structural effects [55, 56]. The PTM-rich regions are predicted to be highly dynamic as they have one droplet-promoting region and two aggregation hot spots. The functions and role of droplet-promoting regions and aggregation hot spots are not fully understood, but they may play a role in regulating the concentration of proteins in the cell [47]. In addition, there are six regions with cellular state-dependent (i.e., context-dependent) interactions, which likely have highly dynamic behavior associated with PRAME’s function. This is consistent with the established notion that the propensity for the disorder of a protein can vary with cellular conditions [48].

PRAME may promote tumor progression through the inhibition of differentiation, growth arrest, and apoptosis induced by retinoic acid [57]. Through this study, we demonstrate that IDPRs, DOT regions, droplet-promoting regions, and aggregation hot spots are present in PRAME and provide it the ability to undergo complex PPI. These entities may be considered when developing novel immunotherapies or biologics targeting PRAME as they may play a role in the increased metastasis and pathogenesis of Class 1 UM. The presence of highly dynamic intrinsically disordered protein domains and a potentially small interaction network make PRAME an even more attractive target than has previously been described [57, 58].

Our study brings new considerations in the molecular behavior of PRAME. Our group’s and other studies have shown that IDPs and IDPRs are associated with different ocular malignancies, including UM [10, 14, 22, 23]. IDPs, IDPRs, and DOT regions have been considered attractive drug targets for many years [12, 22–24]. There is tremendous potential to apply these same principles to developing immunotherapies directed at PRAME to treat uveal melanoma. PRAME is also expressed in other non-UM malignancies, including cutaneous melanoma, breast carcinoma, non-small cell lung cancer, and leukemia [59]. Therefore, the findings in this study may be applied to those pathologies. Additionally, our group has characterized the association of IDPRs found in a protein associated with an ocular neoplasm to the recently appreciated phenomenon of membrane-less organelles (MLOs) that is driven by liquid–liquid phase separation (LLPS). We demonstrate that PRAME can potentially promote the formation of these MLOs via LLPS. The utility of these findings will need to be researched further.

Among the most intriguing recent developments in the field of the molecular biology of the cell is the recognition of the abundant presence of MLOs. MLOs are subcellular structures formed by proteins and other macromolecules not enclosed by a lipid bilayer. These liquid droplet-like entities are formed through the process LLPS, where parts of the homogeneous solution of a cell separate to form two distinct phases. LLPS represent an ideal stress-response mechanism that allows for the spatio-temporal organization of macromolecular components in living cells and are extremely sensitive to even minute variations of the environment (e.g., ionic strength, pH, temperature, and solute concentration) [60–66]. Our group has become interested in these entities because the fluidity of MLOs is rooted in LLPS, which is thought to be driven by the interaction of intrinsically disordered protein regions of various proteins [60, 61, 67–69]. Although MLOs have multiple physiological functions, aberrant LLPS can be deleterious and are linked to various maladies, including neurodegenerative diseases and cancer [70–72]. In the context of our study, we know that PRAME is upregulated in a subset of Class 1 UM associated with an increased risk of metastasis. It is possible that PRAME’s predicted ability to undergo LLPS and propensity to form droplets may alter immune-associated signaling to dampen tumor regulation and enhance cell turnover.

Our study does not come without its limitations. We used databases and bioinformatics tools, and this approach does not represent a comprehensive nor an exhaustive investigation into the molecular behavior of PRAME. We aimed to analyze the intrinsic disorder qualitatively and quantitatively in PRAME and make a presumption on how intrinsic protein disorder may play a role in the pathogenesis of UM. Further work modeling these disordered segments through molecular dynamic simulations or docking analysis would be the next steps to target these regions further effectively. If these efforts were focused on in-vitro disorder-based drug discovery efforts, it may lead to the most success. Consequently, we could develop a novel immunotherapy targeting the intrinsic disorder in PRAME, which may be identified and eventually translated to direct care of PRAME( +) UM patients. These findings are strictly theoretical; however, our findings establish a necessary biological framework for targeting PRAME.

Through this study, we have demonstrated that intrinsically disordered protein regions are likely involved in functionality of PRAME and could be related to its pathogenesis in UM. Further investigation will be necessary to translate these findings into patient care. Modern drug discovery and therapeutic target development are grounded in structure and order-based assumptions. We are taking a different aim than that paradigm and believe these drug discovery campaigns must also be built on the assumption that proteins have inherent flexibility, thus altering the therapeutic development process. This study represents an exciting take on immunotherapy development. Successful efforts to target the intrinsically disordered-based proteins of PRAME may reduce rates of metastasis and poor outcomes in Class 1 uveal melanomas.

## Conclusion

PRAME plays an important role in tumor biology and has been identified as a biomarker for Class 1 UM. PRAME( +) Class 1 tumors are associated with higher rates of metastasis and, thus, worse prognosis. Our study demonstrates that intrinsically disordered protein regions are present in PRAME. IDPRs in PRAME likely have a role in its functionality and protein–protein interactions. We show that intrinsically disordered protein regions or disorder-to-order transition regions in PRAME are likely important for the functionality of the protein and could be targeted when developing immunotherapies for this protein. Future studies should further validate our findings to better elucidate the role of IDPRs in PRAME for the treatment of Uveal Melanoma.

### Supplementary Information

**Additional file 1: Figure S1.** PRAME amino acid sequence. Shown in FASTA format, the protein sequence represents PRAME canonical form. The protein contains 509 residues.

## Data Availability

Data and materials available upon request.

## Abbreviations

UM: Uveal melanoma
GEP: Gene expression profiling
PRAME: Preferentially expressed antigen in melanoma
BRCA: Breast cancer type 1 susceptibility protein
BAP1: BRCA-associated protein 1
IDPs: Intrinsically disordered proteins
IDPRs: Intrinsically disordered protein regions
UniProt: Universal Protein Resource
PBD: Protein Data Bank Select
RIDAO: Rapid Intrinsic Disorder Analysis Online
PONDR: Predictor of Natural Disorder Regions
PPDR: Percent of predicted disorder residues
ADS: Average disorder score
D^2^P^2^: Database of Disordered Protein Prediction
SCOP: Structural Classification of Proteins
MoRFs: Molecular recognition features
PTMs: Posttranslational modifications
DOT: Disorder-to-order transition
DPRs: Droplet-promoting regions
STRING: Search Tool for the Retrieval of Interacting Genes
LLR: Leucine-rich repeats
RARA: Retinoic acid receptor alpha
MLOs: LLPS propensity (p_LLPS_), membrane-less organelles
LLPS: Liquid–liquid phase separation
CAID: Critical Assessment of protein Intrinsic Disorder prediction

## References

[1] Kaliki S, Shields CL (2017). Uveal melanoma: relatively rare but deadly cancer. Eye (Lond).

[2] Chattopadhyay C, Kim DW, Gombos DS, Oba J, Qin Y, Williams MD, Esmaeli B, Grimm EA, Wargo JA, Woodman SE (2016). Uveal melanoma: From diagnosis to treatment and the science in between. Cancer.

[3] Diener-West M, Reynolds SM, Agugliaro DJ, Caldwell R, Cumming K, Earle JD, Hawkins BS, Hayman JA, Jaiyesimi I, Jampol LM (2005). Development of metastatic disease after enrollment in the COMS trials for treatment of choroidal melanoma: Collaborative Ocular Melanoma Study Group Report No. 26. Arch Ophthalmol.

[4] Onken MD, Worley LA, Ehlers JP, Harbour JW (2004). Gene expression profiling in uveal melanoma reveals two molecular classes and predicts metastatic death. Cancer Res.

[5] Onken MD, Worley LA, Char DH, Augsburger JJ, Correa ZM, Nudleman E, Aaberg TM, Altaweel MM, Bardenstein DS, Finger PT (2012). Collaborative Ocular Oncology Group report number 1: prospective validation of a multi-gene prognostic assay in uveal melanoma. Ophthalmology.

[6] Kaler CJ, Dollar JJ, Cruz AM, Kuznetsoff JN, Sanchez MI, Decatur CL, Licht JD, Smalley KS, Correa ZM, Kurtenbach S (2022). BAP1 loss promotes suppressive tumor immune microenvironment via upregulation of PROS1 in class 2 uveal melanomas. Cancers.

[7] Harbour JW, Onken MD, Roberson ED, Duan S, Cao L, Worley LA, Council ML, Matatall KA, Helms C, Bowcock AM (2010). Frequent mutation of BAP1 in metastasizing uveal melanomas. Science.

[8] Djulbegovic MB, Taylor DJ, Uversky VN, Galor A, Shields CL, Karp CL (2022). Intrinsic Disorder in BAP1 and Its Association with Uveal Melanoma. Genes.

[9] Field MG, Decatur CL, Kurtenbach S, Gezgin G, van der Velden PA, Jager MJ, Kozak KN, Harbour JW (2016). PRAME as an Independent Biomarker for Metastasis in Uveal Melanoma. Clin Cancer Res.

[10] Djulbegovic, M. B.; Uversky, V. N.; Harbour, J. W.; Galor, A.; Karp, C. L. Structural Protein Analysis of Driver Gene Mutations in Conjunctival Melanoma. Genes (Basel) 2021, 12 (10). 10.3390/genes12101625 From NLM Medline. 10.3390/genes12101625 PMC8535873 34681018

[11] van der Lee R, Buljan M, Lang B, Weatheritt RJ, Daughdrill GW, Dunker AK, Fuxreiter M, Gough J, Gsponer J, Jones DT (2014). Classification of intrinsically disordered regions and proteins. Chem Rev.

[12] Peng Z, Yan J, Fan X, Mizianty MJ, Xue B, Wang K, Hu G, Uversky VN, Kurgan L (2015). Exceptionally abundant exceptions: comprehensive characterization of intrinsic disorder in all domains of life. Cell Mol Life Sci.

[13] Uversky VN, Dave V, Iakoucheva LM, Malaney P, Metallo SJ, Pathak RR, Joerger AC (2014). Pathological unfoldomics of uncontrolled chaos: intrinsically disordered proteins and human diseases. Chem Rev.

[14] Uversky VN, Oldfield CJ, Midic U, Xie H, Xue B, Vucetic S, Iakoucheva LM, Obradovic Z, Dunker AK (2009). Unfoldomics of human diseases: linking protein intrinsic disorder with diseases. BMC Genomics.

[15] Uversky VN, Oldfield CJ, Dunker AK (2008). Intrinsically disordered proteins in human diseases: introducing the D2 concept. Annu Rev Biophys.

[16] Trivedi, R.; Nagarajaram, H. A. Intrinsically Disordered Proteins: An Overview. Int J Mol Sci 2022, 23 (22). 10.3390/ijms232214050 From NLM Medline. 10.3390/ijms232214050 PMC9693201 36430530

[17] Babu MM, van der Lee R, de Groot NS, Gsponer J (2011). Intrinsically disordered proteins: regulation and disease. Curr Opin Struct Biol.

[18] UniProt C (2019). UniProt: a worldwide hub of protein knowledge. Nucleic Acids Res.

[19] Varadi M, Anyango S, Deshpande M, Nair S, Natassia C, Yordanova G, Yuan D, Stroe O, Wood G, Laydon A (2022). AlphaFold Protein Structure Database: massively expanding the structural coverage of protein-sequence space with high-accuracy models. Nucleic Acids Res.

[20] Tunyasuvunakool K, Adler J, Wu Z, Green T, Zielinski M, Zidek A, Bridgland A, Cowie A, Meyer C, Laydon A (2021). Highly accurate protein structure prediction for the human proteome. Nature.

[21] Jumper J, Evans R, Pritzel A, Green T, Figurnov M, Ronneberger O, Tunyasuvunakool K, Bates R, Zidek A, Potapenko A (2021). Highly accurate protein structure prediction with AlphaFold. Nature.

[22] Vacic V, Uversky VN, Dunker AK, Lonardi S (2007). Composition Profiler: a tool for discovery and visualization of amino acid composition differences. BMC Bioinformatics.

[23] Peng K, Radivojac P, Vucetic S, Dunker AK, Obradovic Z (2006). Length-dependent prediction of protein intrinsic disorder. BMC Bioinformatics.

[24] Obradovic Z, Peng K, Vucetic S, Radivojac P, Dunker AK (2005). Exploiting heterogeneous sequence properties improves prediction of protein disorder. Proteins.

[25] Peng K, Vucetic S, Radivojac P, Brown CJ, Dunker AK, Obradovic Z (2005). Optimizing long intrinsic disorder predictors with protein evolutionary information. J Bioinform Comput Biol.

[26] Romero P, Obradovic Z, Li X, Garner EC, Brown CJ, Dunker AK (2001). Sequence complexity of disordered protein. Proteins.

[27] Xue B, Dunbrack RL, Williams RW, Dunker AK, Uversky VN (2010). PONDR-FIT: a meta-predictor of intrinsically disordered amino acids. Biochim Biophys Acta.

[28] Meszaros B, Erdos G, Dosztanyi Z (2018). IUPred2A: context-dependent prediction of protein disorder as a function of redox state and protein binding. Nucleic Acids Res.

[29] Hu XS, Nascimento TD, Bender MC, Hall T, Petty S, O'Malley S, Ellwood RP, Kaciroti N, Maslowski E, DaSilva AF (2019). Feasibility of a Real-Time Clinical Augmented Reality and Artificial Intelligence Framework for Pain Detection and Localization From the Brain. J Med Internet Res.

[30] Wang H, Li S, Wang C, Wang Y, Fang J, Liu K (2022). Plasma and Vitreous Metabolomics Profiling of Proliferative Diabetic Retinopathy. Invest Ophthalmol Vis Sci.

[31] Hanson J, Paliwal KK, Litfin T, Zhou Y (2019). SPOT-Disorder 2: Improved Protein Intrinsic Disorder Prediction by Ensembled Deep Learning. Genomics Proteomics Bioinformatics.

[32] Emenecker RJ, Griffith D, Holehouse AS (2021). Metapredict: a fast, accurate, and easy-to-use predictor of consensus disorder and structure. Biophys J.

[33] Rajagopalan K, Mooney SM, Parekh N, Getzenberg RH, Kulkarni P (2011). A majority of the cancer/testis antigens are intrinsically disordered proteins. J Cell Biochem.

[34] Oates ME, Romero P, Ishida T, Ghalwash M, Mizianty MJ, Xue B, Dosztanyi Z, Uversky VN, Obradovic Z, Kurgan L (2013). D(2)P(2): database of disordered protein predictions. Nucleic Acids Res.

[35] Dosztanyi Z, Csizmok V, Tompa P, Simon I (2005). IUPred: web server for the prediction of intrinsically unstructured regions of proteins based on estimated energy content. Bioinformatics.

[36] Ishida T, Kinoshita K (2007). PrDOS: prediction of disordered protein regions from amino acid sequence. Nucleic Acids Res.

[37] Walsh I, Martin AJ, Di Domenico T, Tosatto SC (2012). ESpritz: accurate and fast prediction of protein disorder. Bioinformatics.

[38] Andreeva A, Howorth D, Brenner SE, Hubbard TJ, Chothia C, Murzin AG (2004). SCOP database in 2004: refinements integrate structure and sequence family data. Nucleic Acids Res.

[39] Murzin AG, Brenner SE, Hubbard T, Chothia C (1995). SCOP: a structural classification of proteins database for the investigation of sequences and structures. J Mol Biol.

[40] de Lima Morais DA, Fang H, Rackham OJ, Wilson D, Pethica R, Chothia C, Gough J (2011). SUPERFAMILY 175 including a domain-centric gene ontology method. Nucleic Acids Res.

[41] Paysan-Lafosse T, Blum M, Chuguransky S, Grego T, Pinto BL, Salazar GA, Bileschi ML, Bork P, Bridge A, Colwell L (2022). InterPro in 2022. Nucleic Acids Res.

[42] Meszaros B, Simon I, Dosztanyi Z (2009). Prediction of protein binding regions in disordered proteins. PLoS Comput Biol.

[43] Katuwawala A, Peng Z, Yang J, Kurgan L (2019). Computational Prediction of MoRFs, Short Disorder-to-order Transitioning Protein Binding Regions. Comput Struct Biotechnol J.

[44] Hornbeck PV, Kornhauser JM, Tkachev S, Zhang B, Skrzypek E, Murray B, Latham V, Sullivan M (2012). PhosphoSitePlus: a comprehensive resource for investigating the structure and function of experimentally determined post-translational modifications in man and mouse. Nucleic Acids Res.

[45] Erdos G, Pajkos M, Dosztanyi Z (2021). IUPred3: prediction of protein disorder enhanced with unambiguous experimental annotation and visualization of evolutionary conservation. Nucleic Acids Res.

[46] Erdos G, Dosztanyi Z (2020). Analyzing Protein Disorder with IUPred2A. Curr Protoc Bioinformatics.

[47] Hardenberg M, Horvath A, Ambrus V, Fuxreiter M, Vendruscolo M (2020). Widespread occurrence of the droplet state of proteins in the human proteome. Proc Natl Acad Sci U S A.

[48] Horvath A, Vendruscolo M, Fuxreiter M (2022). Sequence-based Prediction of the Cellular Toxicity Associated with Amyloid Aggregation within Protein Condensates. Biochemistry.

[49] Vendruscolo M, Fuxreiter M (2022). Sequence Determinants of the Aggregation of Proteins Within Condensates Generated by Liquid-liquid Phase Separation. J Mol Biol.

[50] Szklarczyk D, Gable AL, Lyon D, Junge A, Wyder S, Huerta-Cepas J, Simonovic M, Doncheva NT, Morris JH, Bork P (2019). STRING v11: protein-protein association networks with increased coverage, supporting functional discovery in genome-wide experimental datasets. Nucleic Acids Res.

[51] Dayhoff GW, Uversky VN (2022). Rapid prediction and analysis of protein intrinsic disorder. Protein Sci.

[52] Necci M, Piovesan D, Predictors C, DisProt C, Tosatto SCE (2021). Critical assessment of protein intrinsic disorder prediction. Nat Methods.

[53] Hu G, Katuwawala A, Wang K, Wu Z, Ghadermarzi S, Gao J, Kurgan L (2021). flDPnn: Accurate intrinsic disorder prediction with putative propensities of disorder functions. Nat Commun.

[54] Wang S, Ma J, Xu J (2016). AUCpreD: proteome-level protein disorder prediction by AUC-maximized deep convolutional neural fields. Bioinformatics.

[55] Bah A, Forman-Kay JD (2016). Modulation of Intrinsically Disordered Protein Function by Post-translational Modifications. J Biol Chem.

[56] Uversky VN (2019). Protein intrinsic disorder and structure-function continuum. Prog Mol Biol Transl Sci.

[57] Epping MT, Wang L, Edel MJ, Carlee L, Hernandez M, Bernards R (2005). The human tumor antigen PRAME is a dominant repressor of retinoic acid receptor signaling. Cell.

[58] Amir AL, van der Steen DM, van Loenen MM, Hagedoorn RS, de Boer R, Kester MD, de Ru AH, Lugthart GJ, van Kooten C, Hiemstra PS (2011). PRAME-specific Allo-HLA-restricted T cells with potent antitumor reactivity useful for therapeutic T-cell receptor gene transfer. Clin Cancer Res.

[59] Ikeda H, Lethe B, Lehmann F, van Baren N, Baurain JF, de Smet C, Chambost H, Vitale M, Moretta A, Boon T (1997). Characterization of an antigen that is recognized on a melanoma showing partial HLA loss by CTL expressing an NK inhibitory receptor. Immunity.

[60] Fonin AV, Antifeeva IA, Kuznetsova IM, Turoverov KK, Zaslavsky BY, Kulkarni P, Uversky VN (2022). Biological soft matter: intrinsically disordered proteins in liquid-liquid phase separation and biomolecular condensates. Essays Biochem.

[61] Antifeeva IA, Fonin AV, Fefilova AS, Stepanenko OV, Povarova OI, Silonov SA, Kuznetsova IM, Uversky VN, Turoverov KK (2022). Liquid-liquid phase separation as an organizing principle of intracellular space: overview of the evolution of the cell compartmentalization concept. Cell Mol Life Sci.

[62] Nesterov SV, Ilyinsky NS, Uversky VN (2021). Liquid-liquid phase separation as a common organizing principle of intracellular space and biomembranes providing dynamic adaptive responses. Biochim Biophys Acta Mol Cell Res.

[63] Alberti S, Hyman AA (2021). Biomolecular condensates at the nexus of cellular stress, protein aggregation disease and ageing. Nat Rev Mol Cell Biol.

[64] Boeynaems S, Alberti S, Fawzi NL, Mittag T, Polymenidou M, Rousseau F, Schymkowitz J, Shorter J, Wolozin B, Van Den Bosch L (2018). Protein Phase Separation: A New Phase in Cell Biology. Trends Cell Biol.

[65] Shin, Y.; Brangwynne, C. P. Liquid phase condensation in cell physiology and disease. Science 2017, 357 (6357). 10.1126/science.aaf4382 From NLM. 10.1126/science.aaf4382 28935776

[66] Banani SF, Lee HO, Hyman AA, Rosen MK (2017). Biomolecular condensates: organizers of cellular biochemistry. Nat Rev Mol Cell Biol.

[67] Uversky VN (2021). Recent Developments in the Field of Intrinsically Disordered Proteins: Intrinsic Disorder-Based Emergence in Cellular Biology in Light of the Physiological and Pathological Liquid-Liquid Phase Transitions. Annu Rev Biophys.

[68] Brocca, S.; Grandori, R.; Longhi, S.; Uversky, V. Liquid-Liquid Phase Separation by Intrinsically Disordered Protein Regions of Viruses: Roles in Viral Life Cycle and Control of Virus-Host Interactions. Int J Mol Sci 2020, 21 (23). 10.3390/ijms21239045 From NLM. 10.3390/ijms21239045 PMC7730420 33260713

[69] Uversky, V. N.; Finkelstein, A. V. Life in Phases: Intra- and Inter- Molecular Phase Transitions in Protein Solutions. Biomolecules 2019, 9 (12). 10.3390/biom9120842 From NLM. 10.3390/biom9120842 PMC6995567 31817975

[70] Ahmad A, Uversky VN, Khan RH (2022). Aberrant liquid-liquid phase separation and amyloid aggregation of proteins related to neurodegenerative diseases. Int J Biol Macromol.

[71] Darling AL, Shorter J (2021). Combating deleterious phase transitions in neurodegenerative disease. Biochim Biophys Acta Mol Cell Res.

[72] Lu J, Qian J, Xu Z, Yin S, Zhou L, Zheng S, Zhang W (2021). Emerging Roles of Liquid-Liquid Phase Separation in Cancer: From Protein Aggregation to Immune-Associated Signaling. Front Cell Dev Biol.

