# Correction: Intrinsic disorder in PRAME and its role in uveal melanoma

**DOI:** 10.1186/s12964-023-01314-x

**Published:** 2023-10-12

**Authors:** Michael Antonietti, David J. Taylor Gonzalez, Mak Djulbegovic, Guy W. Dayhoff, Vladimir N. Uversky, Carol L. Shields, Carol L. Karp

**Affiliations:** 1 Bascom Palmer Eye Institute, University of Miami, 900 NW 17Th Street, Miami, FL 33136 USA; 2 Department of Chemistry, College of Art and Sciences, University of South Florida, Tampa, FL 33612 USA; 3 Department of Molecular Medicine and USF Health Byrd Alzheimer’s Research Institute, Morsani College of Medicine, University of South Florida, Tampa, FL 33612 USA; 4 Ocular Oncology Service, Wills Eye Hospital, Thomas Jefferson University, Philadelphia, PA USA

**Correction: Cell Commun Signal 21, 222 (2023)**

**https://doi.org/10.1186/s12964-023-01197-y**

After publication of this article [1], the authors reported that the author name ‘David J. Taylor Gonzalez’ was incorrectly written as ‘David J. Taylor’.

The original article [1] has been corrected.
