# Supplementary material for: Intrinsic disorder in PRAME and its role in uveal melanoma
Source: Cell Commun Signal. 2023 Aug 25;21:222. doi: 10.1186/s12964-023-01197-y (PMC10463658; doi:10.1186/s12964-023-01197-y)
Supplement: Supplementary file 2 — Additional file 1: Figure S1. PRAME amino acid sequence. Shown in FASTA format, the protein sequence represents PRAME canonical form. The protein contains 509 residues. [file 12964_2023_1197_MOESM1_ESM.docx]

**Figure S1. PRAME amino acid sequence.** Shown in FASTA format, the protein sequence represents PRAME canonical form. The protein contains 509 residues.

>sp|P78395|PRAME_HUMAN Melanoma antigen preferentially expressed in tumors OS=Homo sapiens OX=9606 GN=PRAME PE=1 SV=1

MERRRLWGSIQSRYISMSVWTSPRRLVELAGQSLLKDEALAIAALELLPRELFPPLFMAAFDGRHSQTLKAMVQAWPFTCLPLGVLMKGQHLHLETFKAVLDGLDVLLAQEVRPRRWKLQVLDLRKNSHQDFWTVWSGNRASLYSFPEPEAAQPMTKKRKVDGLSTEAEQPFIPVEVLVDLFLKEGACDELFSYLIEKVKRKKNVLRLCCKKLKIFAMPMQDIKMILKMVQLDSIEDLEVTCTWKLPTLAKFSPYLGQMINLRRLLLSHIHASSYISPEKEEQYIAQFTSQFLSLQCLQALYVDSLFFLRGRLDQLLRHVMNPLETLSITNCRLSEGDVMHLSQSPSVSQLSVLSLSGVMLTDVSPEPLQALLERASATLQDLVFDECGITDDQLLALLPSLSHCSQLTTLSFYGNSISISALQSLLQHLIGLSNLTHVLYPVPLESYEDIHGTLHLERLAYLHARLRELLCELGRPSMVWLSANPCPHCGDRTFYDPEPILCPCFMPN
